# Association of the lncRNA-GAS5 promoter region rs145204276 polymorphism with sevoflurane maintenance anesthesia outcomes on patients undergoing laparoscopic cholecystectomy

**DOI:** 10.15537/smj.2023.44.2.20220617

**Published:** 2023-02

**Authors:** Panpan Zhang, Zhanming Sha

**Affiliations:** *From the Department of Anesthesiology, Shandong Provincial Third Hospital, Jinan, China.*

**Keywords:** Sevoflurane, oxidative stress injure, lncRNA, polymorphism

## Abstract

**Objectives:**

To further investigate how sevoflurane affects the oxidative stress injury (OSI) in patients undergoing laparoscopic cholecystectomy (LC).

**Methods:**

A prospective cohort study was carried out at Shandong Provincial Third Hospital, Jinan, China on 82 gallstone patients who underwent LC, with sevoflurane maintenance during surgery. Genotyping analysis of the rs145204276 polymorphism was performed using the TaqMan platform. Oxidative stress injury and liver injury parameters were also examined. Lipopolysaccharide (LPS)-induced macrophages, which were challenged with sevoflurane, propofol, or the lncRNA-GAS5 overexpressing plasmid, were used to evaluate the effect of Sevoflurane on lncRNA-GAS5-mediated macrophage polarization.

**Results:**

At TM1 and TM2, the levels of OSI markers and long noncoding (lnc) RNA-GAS5 were not obviously different, whereas at the TM3 time point, these indices were significantly different between the Del-Sevoflurane and Del-Propofol subgroups. These indices were not different between the Ins-sevoflurane and Ins-Propofol subgroups at any time point. Cell-based experiments demonstrated that Sevoflurane could increased the lncRNA-GAS5 level in LPS-induced Del-macrophages (*p*=0.0058), but Propofol did not have this effect (*p*=0.847). Both Sevoflurane and Propofol did not have the effect on lncRNA-GAS5 level in LPS-induced Ins-macrophages (*p*=0.321 and *p*=0.822, respectively).

**Conclusion:**

Sevoflurane maintenance can decrease OSI during LC in the Del genotype of the rs145204276 polymorphism. The Del genotype facilitates lncRNA-GAS5 up-regulation under Sevoflurane exposure and therefore decrease the extent of M1 macrophage polarization.

**L**aparoscopic cholecystectomy (LC) is a common surgical method for the removal of gallstones due to its various advantages, including insignificant trauma, minor postoperative pain, short hospitalization, and few cosmetic scars.^1^ Sevoflurane is a useful anesthetic for elective LC, but its effects vary among individuals.^2^ Laparoscopic cholecystectomy can cause pneumoperitoneum, increase intra-abdominal pressure, cause mesenteric hypoxia in the liver and visceral vessels, and increase oxidative stress injury (OSI). It is clarified that sevoflurane can protect LC-induced OSI, but the molecular mechanism is not clear.^3^

Oxidative stress injury is highly associated with immune system disorders.^4^ A recent study has elaborated that long noncoding (lnc) RNAs (lncRNAs) are crucial for immune function.^5,6^ The newly-identified lncRNA-GAS5 was discovered to affect immune cell functions, such as T cell and macrophage polarization.^7^

The rs145204276 polymorphism is located within the promoter of lncRNA-GAS5, and has been reported that the deletion of an allele of the rs145204276 polymorphism (Del genotype) significantly increases the cancers risks.^8^ It has been shown that rs145204276 could affect the immune system and induce inflammatory responses.^9^ Considering that the immune system is affected by anesthesia, and immune system dysfunction can cause OSI, we suspect that the rs145204276 polymorphism may be associated with the effects of anesthesia. Here, we aimed to further investigate how sevoflurane affects on OSI in patients undergoing LC.

## Methods

This prospective cohort study was carried out between January 2020 and January 2022 at the Shandong Provincial Third Hospital, Jinan, China. This study was carried out in accordance with the Declaration of Helsinki. This study was approved by the ethics committee of Shandong Provincial Third Hospital, and all the patients signed an informed consent form.

We used the Pubmed website to search for prior related publications. We first calculated the sample size requirement by using the Chi-square test procedure of PASS software (degrees of freedom=1; power=0.9; alpha=0.05; W=0.55). Based on meeting the minimum sample size requirements, 82 gallstone patients, who underwent LC, with sevoflurane maintenance during surgery in our hospital were included. Another group consisting of 82 gallstone patients who underwent LC, with propofol maintenance during the surgery was set as the control group. Sevoflurane-maintained patients (n=82) or propofol-maintained patients (n=82) were categorized into Sevoflurane group or propofol group. All patients were treated with a 4-trocar standard laparoscopic technique and completed by the same surgical team. Patients with the following conditions were excluded: i) those who could not self-evaluate their physical condition; ii) those who were diagnosed with a certain mental disease, pregnant and lactating women; iii) those who did not obtain informed consent; and iv) those who were younger than 18 years old.

For anesthesia, the patients were injected with 0.1‐g phenobarbital sodium (Jinyao Amino Acid Co., Ltd, Tianjin, China) and 0.5‐mg atropine (Weideli Co., Ltd, Wuhan, China) 30 minutes (min) before anesthesia induction. General anesthesia was induced with sufentanil (0.3 mg/kg) (Humanwell Pharmaceutical Co., Ltd, Yichang, China), midazolam (0.04 mg/kg) (Humanwell Pharmaceutical Co, Ltd, Yichang, China), and propofol (2 mg/kg) (Weideli Co., Ltd, Wuhan, China). Anesthesia was maintained with sevoflurane (2-3% end‐tidal concentration) (Hengrui Co., Ltd, Lianyungang, China) in sevoflurane group or with propofol (4-6 mg/kg•h) in propofol group, in combination with sufentanil (0.15-0.35 µg/kg•h) during surgery.

We set up 5 time-points (TM1, TM2, TM3, TM4 and TM5). TM1 was the time point before surgery; TM2 was 5 min after anesthesia induction; TM3 was 2 hours (h) after surgery; TM4 and TM5 were 24 h and 48 h after surgery. Blood samples were extracted from patients between TM1 and TM5. Serum in ordinary test tubes was centrifuged at 1500 g for 10 min.

### Genotyping

The lncRNA-GAS5 rs145204276 polymorphism was identified using the TaqMan platform (Thermo Fisher Scientific, Waltham, MA) according to previous reports.^8,10-12^ Patients with the rs145204276 Ins/Ins genotype were allocated to the Ins group, and Ins/Del or Del/Del genotype patients were allocated to the Del group.

### Primary and secondary outcome

Oxidative stress injury indicator Malondialdehyde (MDA), tumor necrosis factor alpha (TNF-α) and interleukin 6 (IL-6), and liver injury indicator alanine aminotransferase (ALT) and aspartate aminotransferase (AST), were set as the primary outcome; mean arterial pressure (MAP) and (heart rate) HR were set as secondary outcomes.

### Malondialdehyde, ALT, AST and cytokines levels determination

Serum MDA levels were determined by the method described in the previous literature.^13^ The serum ALT and AST levels were detected using a Beckman UniCel DxC 800 system (Beckman, Brea, CA).^14^ Iinterleukin-6 and TNF-α levels were measured using enzyme-linked immunosorbent assay (ELISA) kits (Minneapolis, MN, USA).

### Flow cytometry

The peripheral blood monocyte/macrophage M1/M2 polarization was determined by flow cytometry according to a previous report.^15^ We first fully mixed 50ul of the blood sample with 5ul of CD163-PE, CD86-APC and CD68-FITC, and placed them in the dark place at room temperature for 20 min. Subsequently, we added 1 ml of erythrocyte lysis solution (Thermo Fisher Scientific, Waltham, MA), and the retained leukocytes were fixed with 2% paraformaldehyde, and then were performed by flow cytometry analysis. All the above antibodies were purchased from ebioscience (Thermo Fisher Scientific, Waltham, MA).

### Quantitative reverse-transcription polymerase chain reaction (qRT-PCR)

Ribonucleic acids were reverse transcribed into cDNAs using a cDNA Synthesis Kit (TaKaRa, Osaka, Japan), and cDNAs were amplified using the SYBR Premix Ex Taq II Kit (TaKaRa, Osaka, Japan). LncRNA-GAS5 levels were measured using the LightCycle®96 Real-time PCR System (Roche, Basel, Switzerland), with glyceraldehyde-3-phosphate dehydrogenase (GAPDH) as the internal reference.

### Sevoflurane-treated macrophages

Peripheral blood mononuclear cells (PBMCs) were isolated and induced to macrophages according to previous report.^16^ After which they were inoculated in a culture plate for the indicated period and placed in a closed plexiglass box. The air inlet of this plexiglass box was connected to the anesthesia vaporizer, and the air outlet was connected to the gas analyzer. A gas content ratio of 5% CO_2_, 21% O_2_, and 74% N_2_ was initially maintained. Before the experiment, Sevoflurane gas was delivered to the plexiglass box at a gas flow rate of 3 L/min with an anesthetic vaporizer, and sevoflurane content was monitored using a gas analyzer. When Sevoflurane concentration was 5%, the air inlet and outlet were closed, and the cells were treated in a closed plexiglass box in a 37°% incubator for 3 h and then placed in a 37°% incubator for subsequent treatment.

### Propofol-treated macrophages

Propofol (Sigma-Aldrich, Merck, Darmstadt, Germany) was diluted in dimethyl sulfoxide (DMSO) to 600 mM. Propofol-supplemented medium was added to the macrophages cultures at 300 µM for 3 h and then placed in a 37° percent incubator for subsequent treatment.

### Plasmid transfection

The human LncRNA-GAS5 cDNA was synthesized and cloned into the pcDNA3.1 vector (named LncRNA-GAS5-OV). Lipofectamine 2000 reagent (Thermo Fisher Scientific, Waltham, MA) was used to transfect the cells.

### Statistical analysis

Results are expressed as an average ± SD. We checked the normality of all the tested data before statistical analyses. The differences between the 2 groups were determined by Student t-test using SPSS Statistics for Windows, v.24 (IBM Corp., Armonk, N.Y., USA) software, if the data meet the normal distribution. If continuous variables were not normally distributed, the Mann-Whitney U-test was employed. The differences between the 2 groups were compared using Pearson’s Chi-square test for discrete variables. *P* values <0.05 were considered significant.

## Results

### Comparing the clinical features of sevoflurane and propofol groups

The perioperative clinical characteristics of the 2 patient groups are shown in Table 1. Age, body mass index (BMI), average operation time, intraoperative bleeding, American Society of Anesthesiologists (ASA) score, comorbidities rate (hypertension and diabetes), and amount of intravenous fluids provided intraoperatively were not significantly different between the 2 groups (Table 1). Among the 82 patients in the sevoflurane group, 33 cases showed the Ins/Ins genotype (named the Ins-sevoflurane subgroup) and 49 cases showed the Ins/Del or Del/Del genotype (named the Del-sevoflurane subgroup). In the propofol group, 35 cases carried the Ins genotype (Ins-Propofol subgroup) and 47 cases carried the Del genotype (Del-propofol subgroup).

**Table 1:** - Comparing the clinical features in sevoflurane group and propofol group (N=82).

Index	Sevoflurane group	Propofol group	*P*-value
Age	44.4±17.6	43.5±19.1	0.8276
Body mass index	23.8±3.7	22.6±3.9	0.1646
Surgery time (min)	50.2±18.7	50.8±17.6	0.8851
Bleeding volume (mL)	41.1±14.3	42.1±15.3	0.7645
Gender (female/male), n	30/52	35/47	0.4247
ASA score (I/II), n	66/16	62/20	0.4505
Hypertension (yes/no), n	23/59	18/64	0.3672
Diabetes (yes/no), n	19/63	26/56	0.2206
Intraoperative infusion volume (mL)	1324±476	1452±587	0.1271
rs145204276 polymorphism phenotype (Ins/Del), n	33/49	35/47	0.7518

ASA: American Society of Anesthesiologists

### Comparing OSI and liver injury parameters of sevoflurane and propofol groups

Increased levels of MDA and cytokines IL-6 and TNF-α, can reflect the extent of OSI, and OSI during surgery can lead to liver injury shortly after surgery.^4^ Therefore, we tested serum MDA, IL-6, and TNF-α levels in TM1, TM2 and TM3, as well as ALT and AST levels in TM1, TM4, and TM5 as primary outcomes. Here, MDA, IL-6, and TNF-α increased from TM1 to TM2 and TM3, and they were lower in sevoflurane group than that in propofol group (Table 2). Alanine aminotransferase and AST enhanced from TM1 to TM4 and TM5, and they were lower in sevoflurane group than that in propofol group (Table 2). We tested MAP and HR in TM1, TM2, and TM3 as secondary outcomes, and found slight decreases in MAP or HR from TM1 to TM2 and TM3, but the differences did not reach statistical significance. MAP and HR had no statistical differences between the 2 groups at any time point (Table 2). Collectively, these results indicate that sevoflurane maintenance leads to stronger protection against OSI than propofol maintenance, consistent with a previous report.^3^

**Table 2:** - Comparing the primary and secondary outcomes at indicating time point in sevoflurane group and propofol group (N=82).

Index	Sevoflurane group	Propofol group	*P*-value
Primary outcomes			
MDA (µmol/L) at TM1	1.4±0.5	1.5±0.7	0.2941
MDA (µmol/L) at TM2	2.3±0.5*	3.0±0.8*	<0.0001
MDA (µmol/L) at TM3	5.1±0.9*	5.8±1.0*	<0.0001
TNF-α (ng/L) at TM1	2.09±0.75	1.97±0.64	0.2720
TNF-α (ng/L) at TM2	2.87±0.82*	2.90±1.14	0.8468
TNF-α(ng/L) at TM3	3.77±1.36*	4.37±1.25	0.0037
IL-6 (ng/L) at TM1	2.74±1.03	2.83±0.77	0.5271
IL-6 (ng/L) at TM2	3.01±1.12*	3.37±1.09*	0.0386
IL-6 (ng/L) at TM3	4.32±1.33*	4.89±0.88*	0.0015
ALT (U/I) TM1	12.5±3.1	12.7±5.6	0.7776
ALT (U/I) TM4	28.6±4.6*	31.9±6.4*	0.0002
ALT (U/I) TM5	32.3±6.2*	39.9±6.9*	<0.0001
AST (U/I) TM1	24.7±4.4	25.9±5.8	0.1375
AST (U/I) TM4	38.8±6.9*	42.4±7.2*	0.0013
AST (U/I) TM5	44.5±7.0*	49.1±6.3*	<0.0001
Secondary outcomes			
MAP (mmHg) at TM1	93.4±14.3	94.7±15.6	0.5788
MAP (mmHg) at TM2	90.1±10.6	91.7±11.4	0.3534
MAP (mmHg) at TM3	89.6±13.2	90.5±10.1	0.6246
HR (beats/min) at TM1	81.7±14.5	82.1±14.4	0.8595
HR (beats/min) at TM2	78.4±13.6	79.3±14.0	0.6768
HR (beats/min) at TM3	71.7±12.0*	69.4±15.6*	0.2915

^*^ *p*<0.05 vs TM1 (baseline). MDA: malondialdehyde, TNF-α: tumor necrosis factor alpha, IL-6: interleukin 6, ALT: alanine aminotransferase, AST: aspartate aminotransferase, MAP: mean arterial pressure, HR: heart rate, TM1 to TM5: timepoint 1 to timepoint 5

### Association of the rs145204276 polymorphism with OSI in sevoflurane and propofol groups

**Figure 1:**
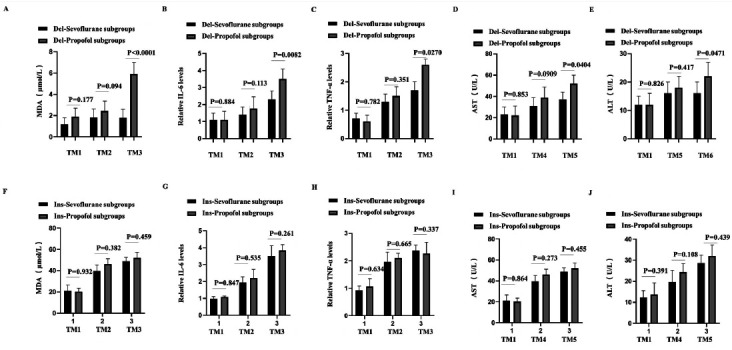
- Association of rs145204276 polymorphism with OSI markers and liver injury markers in sevoflurane group and propofol group. (A-C) Comparing the serum (A) MDA, (B) IL-6 and (C) TNF-α levels between Del-sevoflurane subgroup and Del-propofol subgroup in TM1, TM2 and TM3. (D-E) Comparing the serum (D) AST, and (E) ALT levels between Del-Sevoflurane subgroup and Del-propofol subgroup in TM1, TM4 and TM5. (F-H) Comparing the the serum (F) MDA, (G) IL-6, and (H) TNF-α levels between Ins-sevoflurane subgroup and Ins-propofol subgroup in TM1, TM2 and TM3. (I-J) Comparing the serum (I) AST, and (J) ALT levels between Ins-sevoflurane subgroup and Ins-propofol subgroup in TM1, TM4 and TM5. IL-6 and TNF-α were expressed by fold change compared with TM1 baselines. OSI: oxidative stress injury

The association of the rs145204276 polymorphism with OSI and liver injury indicators at the indicated time points was determined. At the TM3 time point, the levels of MDA, IL-6, and TNF-α in the Del-sevoflurane subgroup were lower than those in the Del-propofol subgroup (Figure 1 A-C). Similarly, at the TM5 time point, the values of ALT and AST in the Del-sevoflurane subgroup were lower than those in the Del-propofol subgroup (Figure 1D-E). There were no significant differences in the levels of MDA, IL-6, TNF-α, ALT and AST between the Ins-Sevoflurane and Ins-Propofol subgroups at any time point (Figure 1 F–J). These results demonstrate that sevoflurane protection against OSI only occurs in patients carrying the Del genotype of the rs145204276 polymorphism.

### Association of the rs145204276 polymorphism with M1/M2 macrophage polarization in sevoflurane and propofol groups

**Figure 2:**
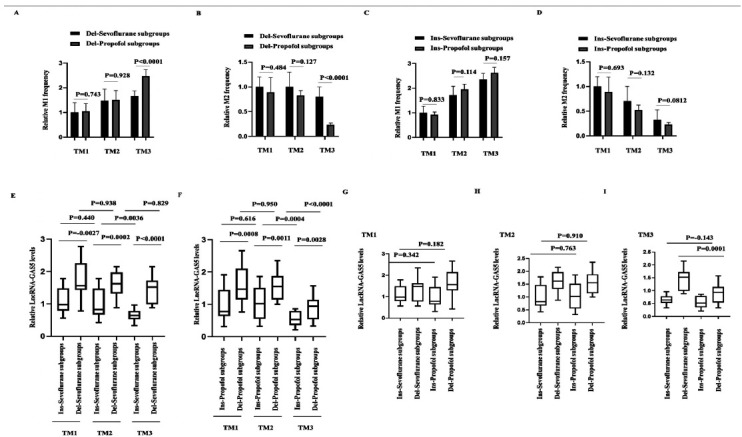
- Association of rs145204276 polymorphism with macrophages M1/M2 polarization and LncRNA-GAS5 levels in sevoflurane group and propofol group. (A-B) Comparing the macrophages (A) M1 polarization and (B) M2 polarization frequency between Del-Sevoflurane subgroup and Del-propofol subgroup in TM1, TM2 and TM3. (C-D) Comparing the macrophages (C) M1 polarization and (D) M2 polarization frequency between Ins-sevoflurane subgroup and Ins-Propofol subgroup in TM1, TM2 and TM3. (E-F) Comparing the macrophages LncRNA-GAS5 levels (E) between Del-Sevoflurane subgroup and Ins-sevoflurane subgroup; and (F) between Del-propofol subgroup and Ins-propofol subgroup in TM1, TM2 and TM3. (G-I) Comparing the macrophages LncRNA-GAS5 levels among Del-sevoflurane subgroup, Ins-sevoflurane subgroup, Del-propofol subgroup and Ins-propofol subgroup in (G) TM1, (H) TM2, and (I) TM3.

Interlukin-6 and TNF-α are mainly secreted by polarized M1 macrophages.^17^ Therefore, we examined the M1/M2 frequency of macrophages between the 2 groups. At TM1 and TM2, macrophage polarization was not significant different between the Del-Sevoflurane and Del-Propofol subgroups. At the TM3 time point, the M1 frequency decreased, while the M2 frequency increased in the Del-sevoflurane subgroup compared with the Del-propofol subgroup (Figure 2A-B). The M1/M2 ratio had no statistical differences between the Ins-sevoflurane and ins-propofol groups at any time point (Figure 2C-D). These results indicate an association between the rs145204276 polymorphism and M1/M2 macrophage polarization, which may play a role in the protective effect of sevoflurane on OSI.

### Association of the rs145204276 polymorphism with lncRNA-GAS5 levels in Sevoflurane and Propofol groups

The rs145204276 polymorphism is located within the promoter of lncRNA-GAS5, suggesting that it is related to the lncRNA-GAS5 level.^8-9^ Furthermore, lncRNA-GAS5 has been reported to regulate the polarization of macrophages.^18-19^ Therefore, we explored the possible association of the rs145204276 polymorphism with the lncRNA-GAS5 level in macrophages of the Sevoflurane and Propofol groups. First, we observed that at the TM1 time point, lncRNA-GAS5 level had no statistical differences between the sevoflurane and propofol groups. However, lncRNA-GAS5 level decreased significantly from TM1 to TM2 and TM3, which gradually recovered at TM4 and TM5 in the 2 groups (Table 3).

**Table 3:** - Comparing the relative LncRNA-GAS5 levels in sevoflurane group and propofol group (N=82).

Index	Sevoflurane group	Propofol group	*P*-value
TM1	1.02±0.33	1.04±0.41	0.7312
TM2	0.66±0.24*	0.71±0.20*	0.1492
TM3	0.42±0.18*	0.59±0.19*	<0.0001
TM4	0.58±0.16*	0.64±0.11*	0.0058
TM5	0.74±0.35*	0.80±0.33*	0.2249

Values are presented as (x±s).

^*^ *p*<0.05 vs TM1 (baseline). TM1 to TM5: timepoint 1 to timepoint 5.

At TM1, TM2, and TM3, the Del-sevoflurane and Del-propofol subgroup showed higher lncRNA-GAS5 levels than the Ins-sevoflurane and Ins-propofol subgroup (Figure 2 E-F). At TM3, the lncRNA-GAS5 level decreased significantly in the Ins-Sevoflurane subgroup compared with that at TM2, but it did not change significantly in the Del-sevoflurane subgroup (Figure 2E). LncRNA-GAS5 level decreased significantly at the TM3 in both the Ins-propofol and Del-propofol subgroups (Figure 2F). Furthermore, at TM1 and TM2, lncRNA-GAS5 level had no statistical differences between the Del-Sevoflurane and Del-propofol subgroups, but the lncRNA-GAS5 level in the Del-sevoflurane subgroup was significantly higher than that in the Del-propofol subgroup at TM3. However, lncRNA-GAS5 level had no statistical differences between the Ins-sevoflurane and Ins-propofol subgroups at any time point (Figure 2 G–I). These results indicate that there is a difference in the regulation of lncRNA-GAS5 levels between sevoflurane and propofol maintenance, which was associated with the rs145204276 polymorphism.

### Regulation of sevoflurane and propofol on macrophage lncRNA-GAS5 levels and M1 macrophage polarization in Ins and Del genotypes

**Figure 3:**
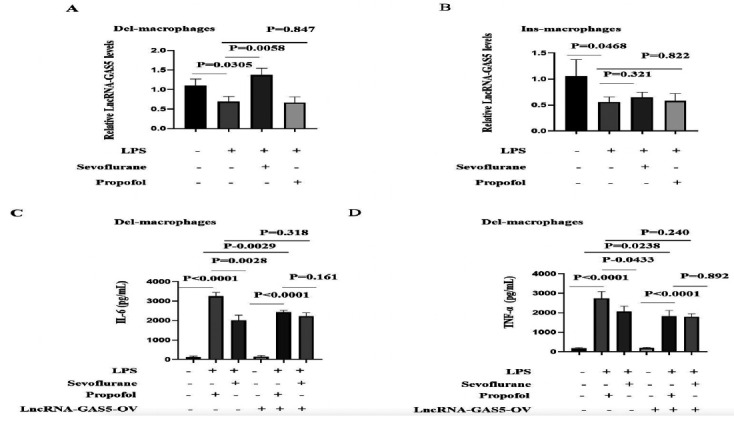
- Regulation of sevoflurane and propofol on blood macrophages long noncoding RNA-GAS5 levels and M1 polarization in Ins genotype and Del genotype. (A) Del genotype (n=3) or (B) Ins genotype (n=3) peripheral blood derived macrophages were pre-exposed by 5% sevoflurane/propofol, followed by lipopolysaccharide (200ng/mL) treatment for 24 hours (h). LncRNA-GAS5 levels were determined by qRT-PCR assay. (C-E) Del genotype peripheral blood derived macrophages were transfected with or without LncRNA-GAS5-OV for 24 h, then were pre-exposed by 5% sevoflurane/propofol, followed by LPS (200ng/mL) treatment for another 24 h. (C) IL-6, and (D) TNF-α levels were determined.

Next, we explored the regulatory mechanism of sevoflurane and propofol on the expression of lncRNA-GAS5 by culturing macrophages in vitro. Three volunteers with the Del genotype and 3 volunteers with the Ins genotype were randomly selected, their whole blood was collected, and macrophages were isolated and cultured in vitro. Cultured macrophages were stimulated with lipopolysaccharide (LPS) to differentiate into the M1 type, followed by treatment with sevoflurane or propofol. We observed that LPS treatment decreased lncRNA-GAS5 levels in both the Del genotype and Ins genotype macrophages. Sevoflurane enhanced lncRNA-GAS5 levels in Del genotype macrophages, whereas propofol failed to affect lncRNA-GAS5 levels. In macrophages with the Ins genotype, the effect of sevoflurane on lncRNA-GAS5 levels was significantly weakened (Figure 3 A-B).

Next, we determined the role of sevoflurane in the regulation of lncRNA-GAS5 on M1 macrophage polarization. We observed that, compared with Propofol, sevoflurane decreased the extent of M1 macrophage polarization, which was reflected by decreases in the M1 macrophage markers IL-6 and TNF-α. These differences were eliminated by lncRNA-GAS5-OV co-exposure, indicating that the decreased lncRNA-GAS5 level was the main reason for sevoflurane-induced M1 macrophage polarization (Figure 3C-D). Collectively, these results demonstrate that in patients with the Del genotype, sevoflurane plays a protective role against inflammation and injury by enhancing the lncRNA-GAS5 level.

## Discussion

Both sevoflurane and propofol have been shown to inhibit proinflammatory cytokine release and OSI.^20-21^ Some investigators have compared the protective effects of sevoflurane and propofol on inflammation and OSI, but the results are contradictory. It is demonstrated that propofol but not sevoflurane prevents OSI in hepatic ischemia/reperfusion injury,^22^ while Xie et al^23^ illustrated that sevoflurane exerts greater protective effects than propofol on hypoxia-reoxygenation injury in cardiomyocytes. We suspect that differences in surgical methods, organs, and patients may be the reasons for these conflicting results. Single nucleotide polymorphisms are important factors in the individualization of patients. We observed that patients under sevoflurane maintenance experienced lower degrees of inflammation and OSI, however, this effect only occurred in the Del genotype of rs145204276, which is located within the lncRNA-GAS5 promoter.

Long noncoding RNA-GAS5, a novel lncRNA, can regulate inflammation and the immune response by interacting with its targets.^24^ A previous study has reported that lower lncRNA-GAS5 expression is associated with tissue damage and inflammatory diseases.^25^ Indeed, inflammation is highly associated with the effects of anesthesia. For example, it has been reported that inflammatory cytokine release is correlated with postoperative Visual Analogu Scale score and anesthetic consumption.^26^ Therefore, we suspect that the rs145204276 polymorphism may modulate the effects of anesthesia and its related OSI by regulating inflammatory cytokine release.

Clinical data indicated higher lncRNA-GAS5 levels and lower proinflammatory cytokine levels in macrophages of the Del-sevoflurane subgroup, which was associated with the polarization of macrophages. In cultured macrophages, sevoflurane increased lncRNA-GAS5 levels in Del genotype macrophages but not in Ins genotype macrophages. Propofol did not alter lncRNA-GAS5 levels in either the Del nor Ins genotype macrophages. Meanwhile, compared with propofol, sevoflurane significantly weakened the polarization of macrophages, which was also related to the up-regulated lncRNA-GAS5 level because after lncRNA-GAS5 overexpression, the changes in the polarization marker levels between the 2 groups were eliminated.

### Study limitations

We did not investigate how the Del genotype of rs145204276 could affect the function of Sevoflurane on lncRNA-GAS5 levels; however, we can provide a reasonable explanation based on our results. Transcription factors can regulate “target” levels by binding to their promoters, and rs145204276 can predictably affect such binding, since it is within the promoter of lncRNA-GAS5. Sevoflurane affects the Del genotype but not the Ins genotype, which suggests that Sevoflurane may regulate lncRNA-GAS5 levels through rs145204276-mediated transcription factors and binding of “targets”. An ongoing study is attempting to explain the role of sevoflurane in the regulation of lncRNA-GAS5. Another limitation is that several factors can affect the OSI in patients undergoing surgical procedures. These factors include nutritional, environmental, and psychological factors, in addition to the stress of surgery. This may also be due to the presentation of acute or chronic cholecystitis. All of these factors play a role in OSI, and might influence OSI and related factors.

In conclusion, sevoflurane can decrease OSI during LC in the Del genotype of the rs145204276 polymorphism. The Del genotype affected the lncRNA-GAS5 level and the associated polarization of M1 macrophages. The implications of this study for future research are that we show that the effects of sevoflurane vary across individuals, and genotyping before surgery will be helpful in the selection of anesthetics. Larger-scale and multi-center studies are needed to eliminate the influence of regional differences or medical conditions.
